# Reproductive Soldier Development Is Controlled by Direct Physical Interactions with Reproductive and Soldier Termites

**DOI:** 10.3390/insects12010076

**Published:** 2021-01-15

**Authors:** Yudai Masuoka, Keigo Nuibe, Naoto Hayase, Takateru Oka, Kiyoto Maekawa

**Affiliations:** 1 Institute of Agrobiological Sciences, NARO (National Agriculture and Food Research Organization), Tsukuba 305-8634, Japan; masuokay781@affrc.go.jp; 2 Graduate School of Science and Engineering, University of Toyama, Toyama 930-8555, Japan; takowasabi1993@gmail.com; 3 School of Science, University of Toyama, Toyama 930-8555, Japan; lucanus1229@gmail.com (N.H.); rutekata1005@gmail.com (T.O.); 4 Faculty of Science, Academic Assembly, University of Toyama, Toyama 930-8555, Japan

**Keywords:** termites, caste differentiation, soldiers, reproductive soldiers, neotenic reproductives, environmental cue

## Abstract

**Simple Summary:**

Generally, in termites, the soldier caste is sterile, and their reproductive organ formation is suppressed. However, in some primitive taxa, neotenic reproductives with soldier-like mandibles, called reproductive soldiers, occasionally appear in the incipient colony. Our first step in understanding the developmental mechanism of this unique caste was to establish efficient conditions to induce reproductive soldiers in *Zootermopsis nevadensis*. Removing both the king and soldiers from an incipient colony strongly stimulated the formation of male reproductive soldiers, which possessed soldier-like mandibles and developed testes. Similarly, high differentiation rates of male reproductive soldiers were observed after king-and-soldier separation treatment using wire mesh. However, no male reproductive soldiers were produced without direct interaction with the queen. These results suggest that reproductive soldier development might be initiated and controlled by direct physical interactions with both reproductives (the king and queen) and soldiers.

**Abstract:**

In eusocial insects (e.g., ants, bees, and termites), the roles of different castes are assigned to different individuals. These castes possess unique phenotypes that are specialized for specific tasks. The acquisition of sterile individuals with specific roles is considered a requirement for social evolution. In termites, the soldier is a sterile caste. In primitive taxa (family Archotermopsidae and Stolotermitidae), however, secondary reproductives (neotenic reproductives) with their mandibles developed into weapons (so-called reproductive soldiers, also termed as soldier-headed reproductives or soldier neotenics) have been reported. To understand the developmental mechanism of this unique caste, it is necessary to understand the environmental cues and developmental processes of reproductive soldiers under natural conditions. Here, we established efficient conditions to induce reproductive soldiers in *Zootermopsis nevadensis*. Male reproductive soldiers frequently developed after the removal of both the king and soldiers from an incipient colony. Similarly, high differentiation rates of male reproductive soldiers were observed after king-and-soldier separation treatment using wire mesh. However, no male reproductive soldiers were produced without direct interaction with the queen. These results suggest that male reproductive soldier development is repressed by direct physical interactions with both the king and soldiers and facilitated by direct physical interaction with the queen.

## 1. Introduction

The complex society of eusocial insects is maintained by the division of labor among castes. In particular, the existence of sterile castes (e.g., the workers in social Hymenoptera and soldiers in termites) that engage in altruistic work without possessing any reproductive abilities is the most crucial characteristic of eusocial insects. However, particularly in termites, the evolutionary mechanisms of sterile castes are still far from fully understood. In termites, the soldier is regarded as the first permanently sterile caste [1,2,3], and they engage only in the defense of their colony [3]. However, secondary reproductives (called “neotenics”) that possess both reproductive organs and weapons appear occasionally in several species of primitive termite taxa (family Archotermopsidae and Stolotermitidae) [4,5,6]. These individuals are so-called reproductive soldiers (also termed as soldier-headed reproductives or soldier neotenics [7]), and males are more frequently observed than females under natural conditions [4,5,6]. These reproductive soldiers are discriminated from other soldiers by their slightly rounded head capsules, different ratios of mandible length to labrum length, and well-developed gonads [4,5,6]. Based on behavioral analysis of *Zootermopsis nevadensis*, reproductive soldiers were suggested to be an adaptive caste for intercolonial battles between young colonies [4]. Male reproductive soldiers were observed when the male primary reproductive (i.e., the king) was removed from a colony of *Z. nevadensis* [6], although the effect of soldier existence and the conditions necessary for the development of female reproductive soldiers were not investigated. Moreover, reproductive soldier-like individuals were artificially induced by the juvenile hormone (JH) analog treatment of *Z. nevadensis* nymphs [8]. However, the differentiation of reproductive soldiers is rare under natural conditions, and it is impossible to identify these individuals before they molt into reproductive soldiers. Thus, the environmental cues that promote the differentiation of this unique caste remain unknown.

The evolution and existence of reproductives and soldiers, both of which undergo caste-specific morphogenesis during molt, are the most important aspects of termite sociobiology. The differentiation of reproductives and soldiers is generally thought to be inhibited by the presence of the same caste and promoted by the absence of various castes [9]. Moreover, the differentiation of reproductives seems to be regulated in a sex-specific manner. For example, experiments on the damp-wood termite, *Hodotermopsis sjostedti* (family Archotermopsidae), clearly show that the presence of female neotenics suppresses the differentiation of further female neotenics, whereas the presence of male neotenics promotes the differentiation of female neotenics [10]. Consequently, the differentiation of male (or female) reproductive soldiers may be affected by the presence of both male (or female) reproductives and soldiers. To clarify this, we used incipient colonies of *Z. nevadensis* in which the development of the first soldier in the colony had been identified [11,12]. In incipient colonies of this species, larvae above the second instar are engaged in worker tasks (thus called “workers” in this study), and the first soldier is differentiated from the oldest third-instar larva [11]. First, we observed the effects of the absence of (1) the king only and (2) the king and soldiers, on the development of male reproductive soldiers. We performed the king and soldier removal experiment over three different periods: (1) before and just after the emergence of the earliest soldier and (2) one month and (3) two months after the emergence of the earliest soldier. We observed the morphological characteristics and mating behavior of specific individuals. Second, we attempted to induce female reproductive soldiers using the most effective methods for the development of male reproductive soldiers to identify any sexual differences. Lastly, we performed separation treatments using wire mesh to determine the effects of direct and indirect physical contact with reproductives and soldiers on reproductive soldier development. Based on the results obtained, we discuss the environmental factors affecting the production of reproductive soldiers, and the specific morphology related to the role of this unique caste.

## 2. Materials and Methods

### 2.1. Termites

To set up incipient colonies, 13 mature colonies of *Z. nevadensis* were collected from laurel forests in Hyogo Prefecture, Japan, in January, April, and September 2016–2019. This population was introduced to this area in the 1990s, and identified as hybrids between the two subspecies, *Z*. *n. nevadensis* and *Z*. *n. nuttingi* [13]. The collected colonies were kept in plastic cases at 25 °C in constant darkness. Newly molted alates (male and female winged individuals) from 11 different colonies were paired in 60-mm plastic cases containing wood chips and kept at 25 °C in constant darkness. In accordance with previous studies [11,12], 302 incipient colonies were founded by mating male and female alates in 60-mm plastic dishes containing wood chips, and these colonies were kept at approximately 25 °C in constant darkness.

### 2.2. Observation of Soldier and Reproductive Soldier Differentiation

To induce male reproductive soldiers, the removal of both the king and soldiers, or only the king, was performed at three time points: (1) just after the first soldier emerged (*n* = 21 and 63 colonies, respectively); (2) one month after the first soldier emerged (*n* = 15 and 16 colonies, respectively); and (3) two months after the first soldier emerged (*n* = 47 and 47 colonies, respectively). The induction rates of reproductive soldiers under seven conditions, including a control without any removal of individuals (*n* = 14 colonies), were measured three months after the removal treatments.

To induce female reproductive soldiers, queen and soldier removal treatment was performed two months after the first soldier emerged, which was the most effective period for the development of male reproductive soldiers (see Section Results) (*n* = 29 colonies). Statistical analysis was performed using Ryan’s test to compare each induction condition in R ver. 3.1.2 [14]. The sexual differences in caste differentiation were compared statistically using Fisher’s exact test in R ver. 3.1.2 [14].

### 2.3. Observation of Mating Behavior

A newly emerged reproductive soldier and a queen from the same incipient colony were kept in a 60-mm glass petri dish with filter paper (*n* = 4 pairs). The behavior of both individuals was recorded for three weeks (3 h/day) from the emergence of reproductive soldiers. Observations were carried out under red lighting using a CX1 digital camera (Rikoh, Tokyo, Japan). When the queen laid eggs, only the eggs were returned to the original incipient colony to confirm hatching.

### 2.4. Morphometry of Emerged Individuals

The individuals used for morphometry were fixed in FAA solution (ethanol:formalin:acetic acid = 16:6:1) for 24 h and preserved in 70% ethanol. In accordance with previous studies [6,8,15], 11 traits (left mandible length, head length, head width, pronotum length, pronotum width, mesonotum width, metanotum width, hind femur length, hind femur width, hind tibia length, and maximum testis width) of four castes (reproductive soldiers, soldiers, third/fourth instar larvae (workers in this study), and neotenics) were measured. The testes were carefully isolated by dissection according to the methods previously described in the literature [8]. Measurements were performed using an SZX10 stereo microscope and 3CCD digital camera XD250-2D (Olympus, Tokyo, Japan). Principal component analysis was performed based on these 11 measurements using Mac Multivariate Statistical Analysis ver. 1.0a (Esumi, Tokyo, Japan). One-way analysis of variance (ANOVA) and Tukey–Kramer test were performed for comparison among each caste using Mac Statistical Analysis ver. 2.0 (Esumi). From the captured images of the neotenic and worker head capsules, the average color properties of the head-capsule area were detected using the color picker tool of Adobe Photoshop CS 7.0 (Adobe Systems Inc., San Jose, CA, USA). In accordance with a previous study [16], the color properties of each individual were evaluated as the average value of 10 randomly chosen points, using the hue angle, saturation, and brightness color model. One-way ANOVA followed by Tukey–Kramer test or Welch’s *t*-test was performed for comparisons using Mac Statistical Analysis ver. 2.0 (Esumi).

### 2.5. Effects of Direct or Indirect Contact with Reproductives and Soldiers

According to methods previously outlined in the literature [10], 60-mm plastic dishes were divided into two semi-circular arenas using a double metal-wire mesh (mesh diameter = 1 mm). First, to clarify the effects of direct vs. indirect contact with the king and soldiers, incipient colonies (two months after first-soldier emergence) were prepared. A king and a soldier were placed in one of the two arenas, and the other arena contained a queen and workers (more than one) (*n* = 19 colonies). The king/soldier arena also contained one female worker as a helper. For the control treatment, individuals of all castes were placed in undivided dishes (*n* = 14 colonies). Second, to determine whether direct contact with a queen was needed for male reproductive soldier development, incipient colonies (two months after emergence of the first soldier) were prepared, and both the king and soldiers were removed from each colony. In one of the two arenas, a queen was placed with either male workers (more than one) or female workers (more than one), and the other arena contained female workers (more than one) or male workers (more than one), respectively (*n* = 10 and 12 colonies, respectively). The induction rates of male reproductive soldiers were measured six months after the treatments. Statistical analysis was performed using Fisher’s exact test in R ver. 3.1.2 [14].

## 3. Results

### 3.1. Effects of King and Soldier Removal Treatment on Reproductive Soldier Development

**Figure 1:**
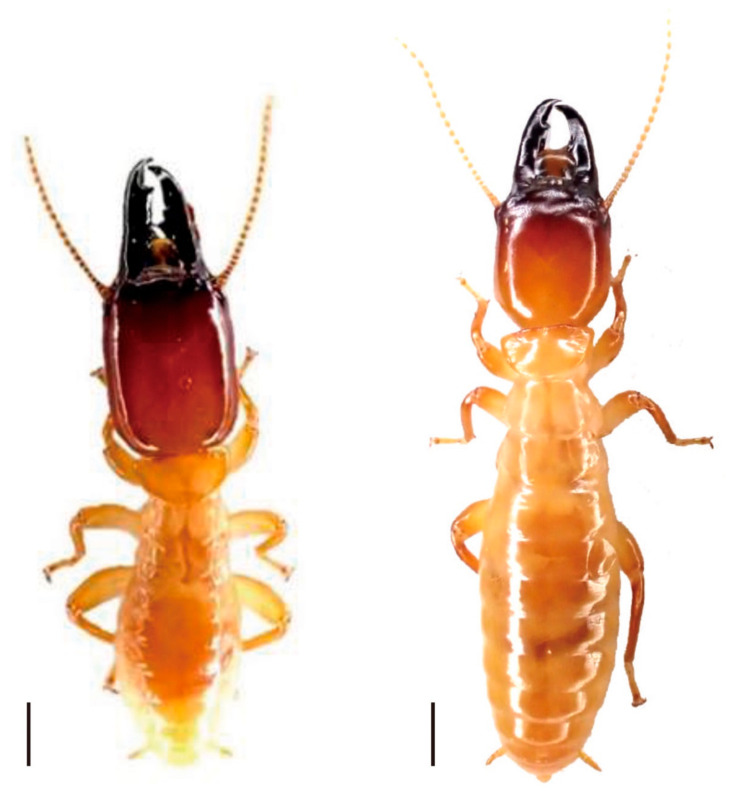
Male phenotypes of the normal soldier (**left**) and reproductive soldier (**right**) observed in an incipient colony of *Z. nevadensis*. Reproductive soldiers possess rounded heads, smaller mandibles, and larger abdomens than did normal soldiers. Scale bar indicates 1 mm.

Male reproductive soldiers were observed under all experimental conditions except for the control treatment (Figure 1). The emergence rates differed depending on the timing of removal (Table 1). When removals were performed at the time of first-soldier emergence, male reproductive soldiers were observed in 4.8% of the king removal colonies (1/21) and 3.2% of the king-and-soldier removal colonies (2/63). These reproductive soldiers differentiated about three months after treatment. In contrast, in treatments one and two months after first-soldier emergence, male reproductive soldiers were differentiated in many more colonies (Table 1). In particular, high differentiation rates were observed in 51.1% of the colonies that underwent king-and-soldier removal treatment two months after first-soldier emergence (24/47; significantly higher rates, Ryan’s test, *p* < 0.05). These individuals differentiated about two months after treatment. Normal male neotenics were observed in 63.6% (7/11) and 44.9% (23/61) of the colonies treated one and two months after first-soldier emergence, in which reproductive soldiers did not develop, respectively. These neotenics possessed a yellowish cuticle on the head capsule, and their color components (saturation and brightness) were significantly different from those of workers (Welch’s *t*-test, *p* < 0.05; Figure S1A).

### 3.2. Behavior and Morphology of Reproductive Soldiers

Mating behavior between a queen and reproductive soldier was recorded in 3/4 pairs examined (Video S1). Two mated queens laid eggs and hatching was observed, although there was a possibility that the queen had mated with the king before the experiment.

**Figure 2:**
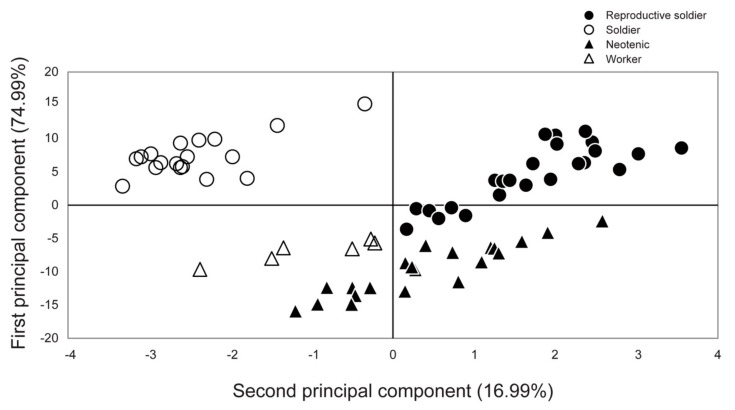
Principal component analysis based on the external morphological measurements of each caste. All individuals examined were males. The contribution ratio of each principal component is indicated in parentheses. Based on the eigenvectors calculated, the first and second principal components mainly indicate differences in head/mandible sizes and testis sizes, respectively. Black and white points indicate reproductive and sterile castes, respectively. Black circles, reproductive soldiers; white circles, soldiers; black triangles, neotenics; white triangles, workers.

**Figure 3:**
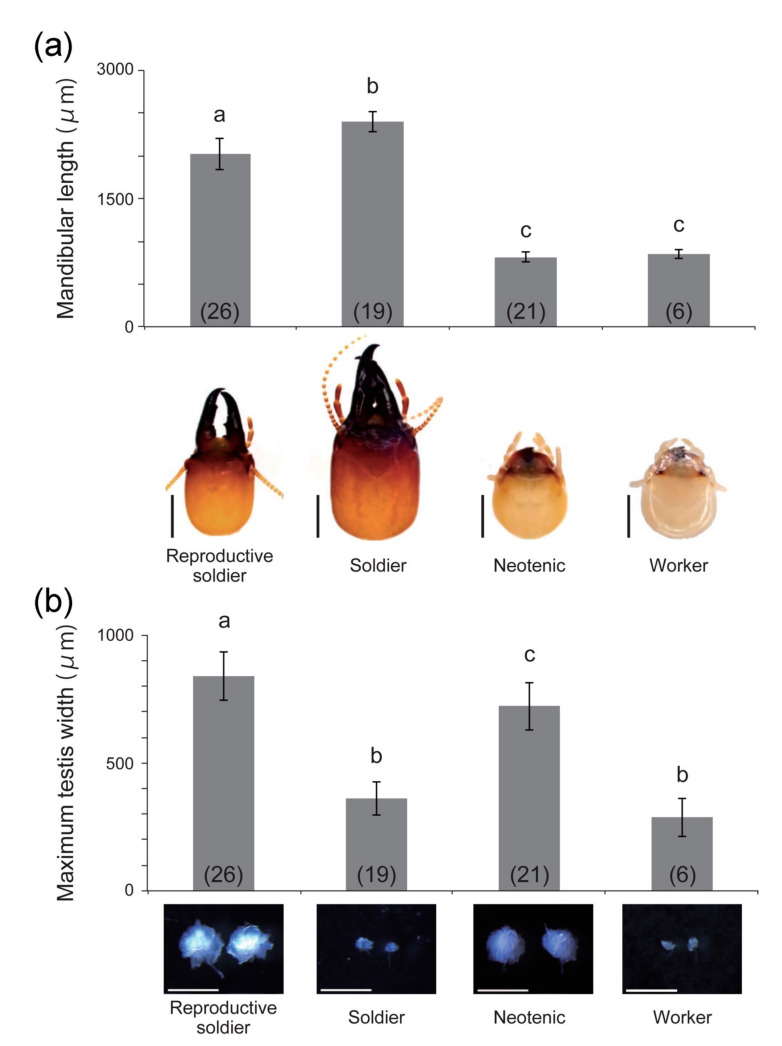
Left mandible (**a**) and testis (**b**) sizes (mean ± S.D.) of each male caste (n = 6–26). The bottom photographs show the head capsule of each caste (**a**) and the testes dissected from individuals (**b**). The numbers of individuals examined are indicated in parentheses. Different letters above the bars denote significant differences (One-way ANOVA followed by Tukey–Kramer test, *p* < 0.05). Scale bars indicate 1 mm (**a**) and 500 μm (**b**).

In principal component analysis, the reproductive soldiers were plotted separately from the other three castes (Figure 2). Comparative analysis showed that the first principal component scores of the reproductive soldiers were significantly different from those of the neotenics and workers (Tukey–Kramer test, *p* < 0.05; Figure S2A). The second principal component scores of the reproductive soldiers were significantly different from those of the other three castes (Tukey–Kramer test, *p* < 0.05; Figure S2B). The mandible lengths of the reproductive soldiers were significantly shorter than those of the soldiers, but longer than those of the neotenics and workers (Figure 3a). However, their testis widths were the largest among the castes (Figure 3b).

We managed to observe some individuals before they molted into soldiers and reproductive soldiers (both of which involved two molts from the worker stage). The external morphologies of the intermediate stages, presoldier, and pre-reproductive soldier were compared, and mandibular lengths and head capsule sizes were significantly different between the two (Figure S3A,B). However, the head sizes of gut-purged workers (before molting into intermediates) were not significantly different between the two groups (Figure S3C). The lengths of the gut-purged periods before molting into each intermediate (presoldier or pre-reproductive soldier) and that of the intermediate periods were also not significantly different (Figure S3D).

### 3.3. Effects of Queen and Soldier Removal Treatment on Reproductive Soldier Development

**Figure 4:**
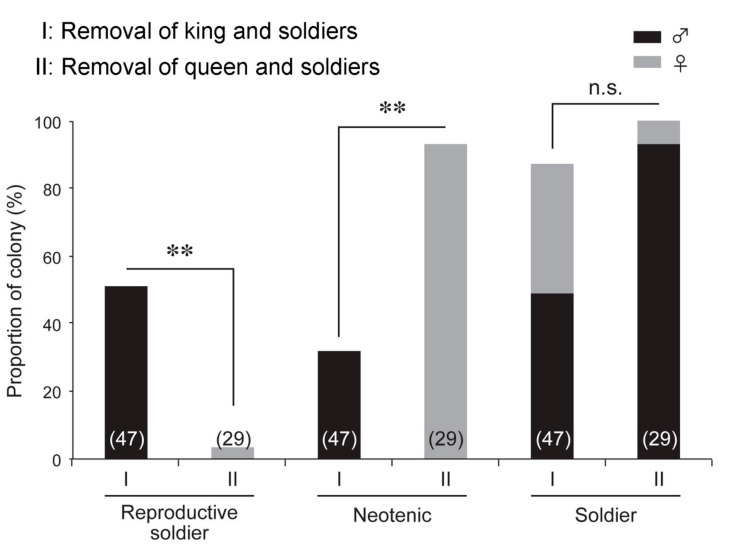
Colony proportions of three castes (reproductive soldiers, neotenics, and soldiers) after the removal of the king and soldiers (I) or the queen and soldiers (II). The numbers of colonies examined are indicated in parentheses. Asterisks denote significant differences (Fisher’s exact test, **p < 0.01, not significant (n.s.)).

The queen and soldier removal experiments were performed two months after the emergence of the first soldier, which was the most effective period for the development of male reproductive soldiers (Table 1). Unexpectedly, female reproductive soldiers were observed in only 1/29 colonies examined (3.4%; Figure 4). However, female neotenics appeared in almost all the colonies examined (27/29) (93.1%; Figure 4). These neotenics had a yellowish cuticle color on the head capsule, similar to the male neotenics, and their color components (hue angle and saturation) were significantly different from those of workers (Welch’s *t*-test, *p* < 0.05; Figure S1B). Although the normal soldiers were newly differentiated in both experiments, the soldiers were strongly biased toward males in the colonies with the queen and soldiers removed (27/29) (93.1%; Figure 4). A previous study showed that the sex ratio of the first soldiers was 9:10 (female:male) in normal colonies [11].

### 3.4. Effects of Direct Contact with Reproductives and Soldiers

**Figure 5:**
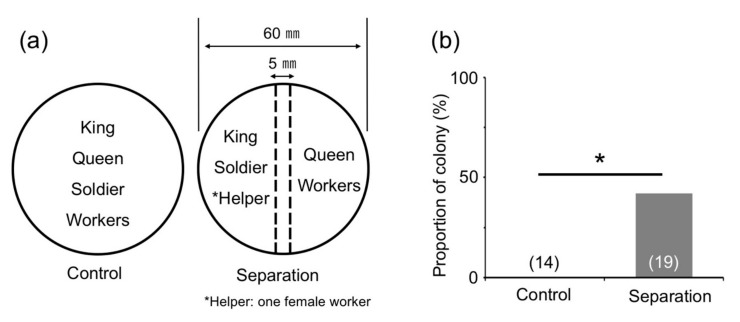
Effects of king-and-soldier separation on male reproductive soldier development. (**a**) Incipient colonies two months after the emergence of the first soldier were prepared for analysis. Broken lines indicate metal-wire mesh. (**b**) Male reproductive soldier development was only observed in the separation treatment. The numbers of colonies examined are indicated in parentheses. Asterisk denotes significant differences (Fisher’s exact test, * *p* < 0.05).

**Figure 6:**
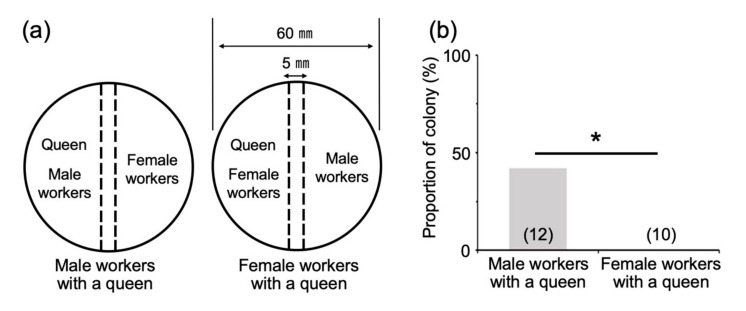
Effects of queen separation on male reproductive soldier development. (**a**) Incipient colonies two months after the emergence of the first soldier were prepared for the analysis. Broken lines indicate metal-wire mesh. (**b**) Male reproductive soldier development was only observed in the male-workers-with-queen treatment. The numbers of colonies examined are indicated in parentheses. Asterisk denotes significant differences (Fisher’s exact test, * *p* < 0.05).

We confirmed that the number of workers in each colony two months after the emergence of the first soldier was similar to that when a presoldier appeared (about 10 individuals [17]). Using these colonies, king-and-soldier separation treatment was performed using wire mesh (Figure 5a), and male reproductive soldiers were observed in 42.1% of the colonies (8/19; Figure 5b). This proportion was significantly higher than that in the control treatment (0/14; Figure 5b). Next, after the removal of the king and soldiers, from each colony two months after the emergence of the first soldier, queen separation treatment was performed (Figure 6a). Male reproductive soldiers were observed in the arena containing male workers and a queen (41.7%, 5/12; Figure 6b). The male workers separated from the queen by the mesh never developed into reproductive soldiers (0/10; Figure 6b).

## 4. Discussion

### 4.1. Morphological Changes during Reproductive Soldier Differentiation

The weapon (mandible) and testis sizes of the reproductive soldiers were clearly different from those of normal soldiers. Male reproductive soldiers possessed smaller head capsules and mandibles but had well-developed testes, as shown in previous studies [4,5,6]. Of note, differences in weapon sizes between soldiers and reproductive soldiers were also observed at the intermediate stages, whereas there were no differences in the head sizes of the workers that molted into each intermediate type. Generally, in insects, there is a trade-off between weapon size and reproductive organ size [18,19,20]. Furthermore, such trade-offs might be mediated by JH in stalk-eyed flies [19] and termites [21,22]. It is interesting to note that the head phenotypes of the reproductive soldiers, with their small mandibles, were highly similar to those of molted soldiers after RNAi-mediated knockdown of the JH receptor gene [12]. Moreover, gonad development was promoted by JH analog treatment in reproductive soldier-like individuals artificially induced from nymphs [8]. These results suggest that the formation of reproductive soldiers is affected by a trade-off relationship mediated by JH. To clarify this possibility, detailed developmental comparisons among reproductive soldiers, normal soldiers, and neotenics should be performed.

### 4.2. Sexual Differences in Reproductive Soldier Differentiation

In this study, the removal of the king and soldiers strongly induced the differentiation of male reproductive soldiers. In contrast, removal of the queen and soldiers did not strongly induce the differentiation of female reproductive soldiers. In the latter treatment, female neotenics appeared instead, in almost all colonies examined. Moreover, the newly emerged soldiers were strongly biased toward males in these colonies. Termite female reproductives (queens and neotenics) normally possess distinctly developed ovaries. In contrast, even the immature testes of soldiers possess the ability to generate sperm in some primitive termite species, such as *H. sjostedti* [23]. A well-developed ovary is crucial for successful reproduction in female neotenics, whereas even small testes may be enough for successful reproduction in male neotenics and reproductive soldiers. We suggest that these differences in physiological constraints between the sexes are related to the tendency of males to differentiate into reproductive soldiers. However, the proximate factors explaining why the combination of two castes (i.e., soldier and neotenic) is possible in males but not in females are still unclear. Further analyses of the molecular mechanisms underlying sexual maturity in termites are needed to clarify this issue. Moreover, the phylogenetic factors related to reproductive soldier development should also be explored, as they are frequently observed only in the males of Archotermopsid species (for example, in *Z. nevadensis*, but not in *H. sjostedti*).

### 4.3. Environmental Cues for Male Reproductive Soldier Differentiation

Previous studies showed that neotenic and soldier differentiation in some species was inhibited by the existence of neotenics and soldiers, respectively, via direct or indirect interactions [10,24,25,26]. In an incipient colony, all members possess the opportunity to encounter all other individuals, and pheromonal information diffuses easily throughout the whole nest, probably because of the relatively small spatial cavity and a small number of colony members. In the present study, male reproductive soldiers were more frequently observed after the removal of the king and soldiers than after the removal of only the king. These results indicate that an absence of information from both the king and soldiers is required for the effective differentiation of male reproductive soldiers. Moreover, the separation treatment using wire mesh suggests that the inhibiting factor(s) from the king and soldiers are non-volatile substances transmitted by direct physical contact. According to previous studies on other species [26], in response to the absence of a king, a queen may promote the differentiation of male (neotenic) reproductives. In the present study, the male workers without direct interactions with the queen never differentiated into reproductive soldiers. Thus, the promoting factor(s) of male reproductive soldier development may also be derived from the queen via direct physical contact. There is a possibility that these non-volatile pheromonal substances are cuticular hydrocarbons and/or specific cuticular hydrocarbon profiles, such as the king and queen recognition pheromones identified in other species [27]. Moreover, a previous study shows that soldier-destined larvae are frequently engaged in proctodeal trophallaxis and allogrooming behaviors with reproductives in *Z. nevadensis* incipient colonies [11]. Promoting factor(s) could be delivered via physical interaction during these social behaviors.

## 5. Conclusions

We established efficient induction conditions for reproductive soldiers in *Z. nevadensis*. Using these conditions, we verified the environmental cues for reproductive soldier development. The male reproductive soldiers induced by the king and soldier removal treatment had soldier-like mandibles and well-developed testes, and mating behaviors with the queen were observed. Separation treatment using wire mesh suggests that (1) the physical absence of both king and soldiers and (2) direct physical interaction with the queen are crucial environmental cues for male reproductive soldier development. Therefore, reproductive soldier development may be initiated and controlled by non-volatile pheromonal substances from reproductives (the king and queen) and soldiers.

## Figures and Tables

**Table 1:** Rates of colonies with reproductive soldiers or neotenics after each treatment.

King Removal Timing	Soldier Removal Timing	Colony Number ^1^	Reproductive Soldier Development ^2^	Days Until Reproductive Soldier Development (n)	Normal Male Neotenic Development
-	-	14	0% (0/14) a	-	0% (0/14)
First-soldier emergence	-	21	4.8% (1/21) a	90.0 (1)	N/A ^3^
First-soldier emergence	First-soldier emergence	63	3.2% (2/63) a	91.5 ± 21.5 (2)	N/A ^3^
+1 month	-	15	33.3% (5/15) ab	89.4 ± 34.9 (5)	30% (3/10) ^4^
+1 month	+1 month	16	25.0% (4/16) ab	51.0 ± 13.0 (4)	60% (6/10) ^4^
+2 months	-	47	19.1% (9/47) a	58.9 ± 22.1 (9)	31.9% (15/47)
+2 months	+2 months	47	51.1% (24/47) b	61.2 ± 19.4 (11)	31.9% (15/47)

^1^ Number of colonies examined for observation of reproductive soldiers. ^2^ Different letters indicate significant differences (Ryan’s test, *p* < 0.05). ^3^ Not applicable. ^4^ Randomly selected 10 colonies were investigated.

## Supplementary Materials

The following materials are available online at https://www.mdpi.com/2075-4450/12/1/76/s1, Figure S1: Head color features of workers and neotenics, Figure S2: The first (A) and second (B) principal component scores (mean ± S.D.) of each caste, Features of the intermediate stages of soldiers and reproductive soldiers. Intermediate stage of normal solider (A, left, pre-soldier) and reproductive soldier (A, right, pre-reproductive soldier). The sizes of the mandibles and head capsules of the intermediate stages (B), head widths of gut-purged workers before the intermediate stages (C), lengths of gut-purged periods of workers before molting to intermediate stage, and the intermediate periods (D) are compared between the two groups, Video S1: Mating behavior between the queen and male reproductive soldier.
