# Supplementary material for: Reproductive Soldier Development Is Controlled by Direct Physical Interactions with Reproductive and Soldier Termites
Source: Insects. 2021 Jan 15;12(1):76. doi: 10.3390/insects12010076 (PMC7830014; doi:10.3390/insects12010076)
Supplement: Supplementary file 1 [file insects-12-00076-s001.zip › supplementary_materials/SuppleFigs_Caption.docx]

**Supplementary Figure Captions**

**Fig. S1.**

Head color features of workers and neotenics. The hue angle, saturation, and brightness (HSB) values (mean ± S.D.) of males (A) and females (B) are shown. The numbers of individuals examined are indicated in parentheses. Asterisks denote significant differences (Welch’s t-test, P < 0.05).

**Fig. S2.**

The first (A) and second (B) principal component scores (mean ± S.D.) of each caste. The numbers of individuals examined are indicated in parentheses. Different letters above the bars denote significant differences (One-way ANOVA followed by Tukey-Kramer’s test, P < 0.05).

**Fig. S3.**

Features of the intermediate stages of soldiers and reproductive soldiers. Intermediate stage of normal solider (A, left, pre-soldier) and reproductive soldier (A, right, pre-reproductive soldier). The sizes of the mandibles and head capsules of the intermediate stages (B), head widths of gut-purged workers before the intermediate stages (C), lengths of gut-purged periods of workers before molting to intermediate stage, and the intermediate periods (D) are compared between the two groups. The numbers of individuals examined are indicated in parentheses. Asterisks denote significant differences (Welch’s t-test, P < 0.05). Scale bar indicates 1 mm.

**Movie 1.**

Mating behavior between a queen and a male reproductive soldier. This queen was observed laying eggs 6 days after the observation, and hatching was observed 15 days after oviposition.
