# Supplementary figures and images for: Reproductive Soldier Development Is Controlled by Direct Physical Interactions with Reproductive and Soldier Termites
Source: Insects. 2021 Jan 15;12(1):76. doi: 10.3390/insects12010076 (PMC7830014; doi:10.3390/insects12010076)

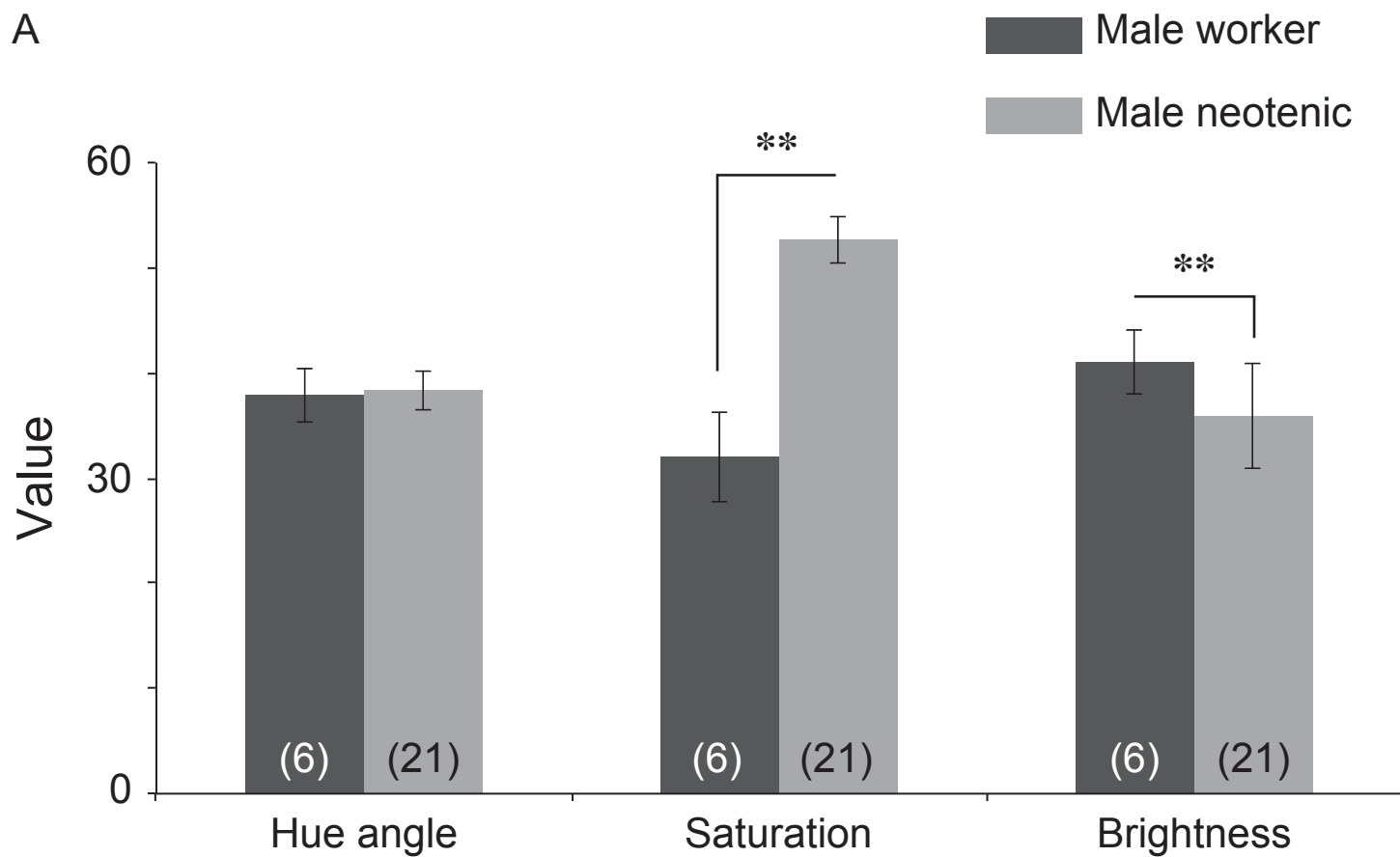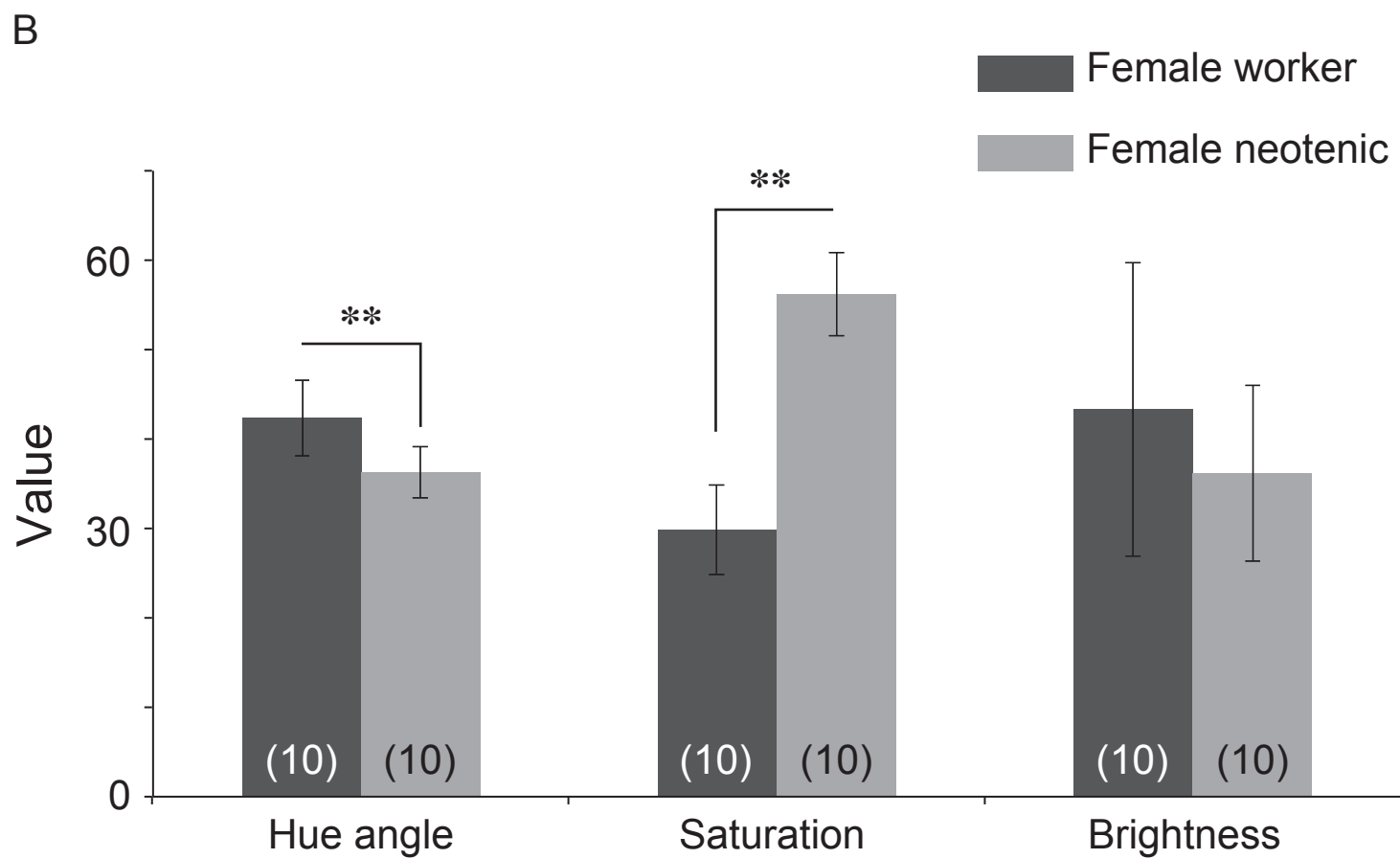

Fig. S1

A

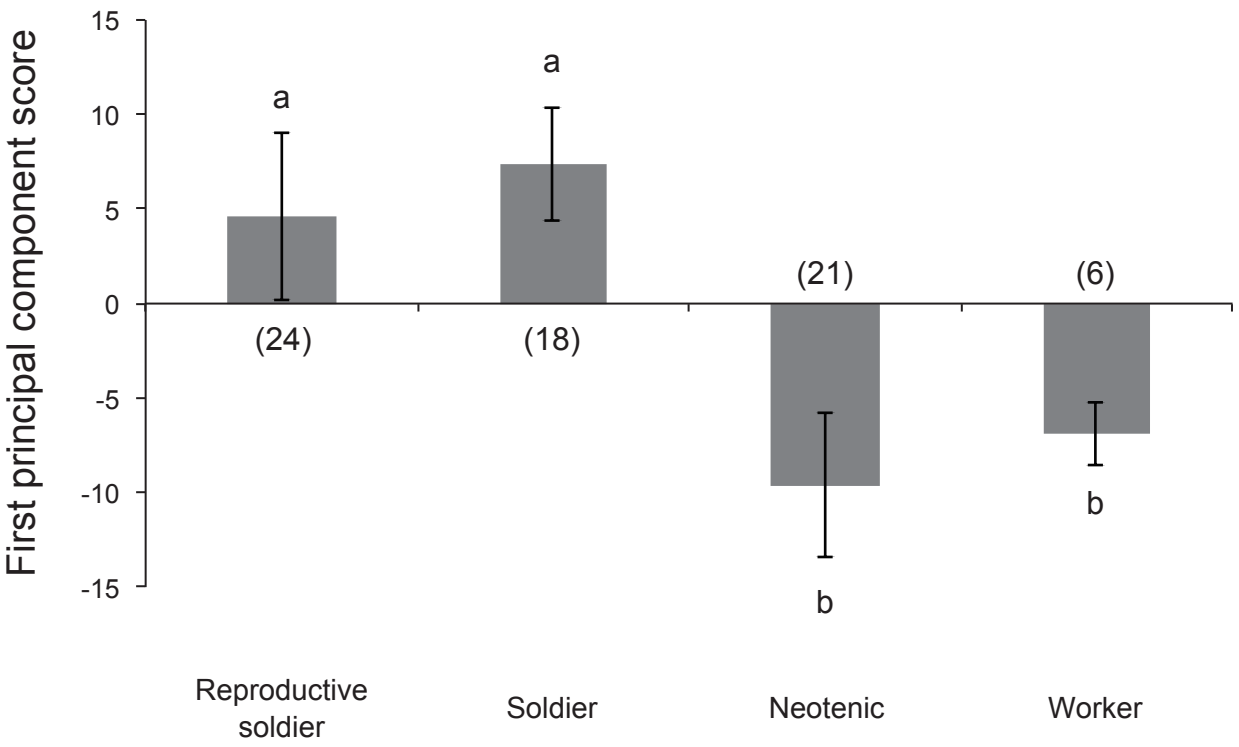

B

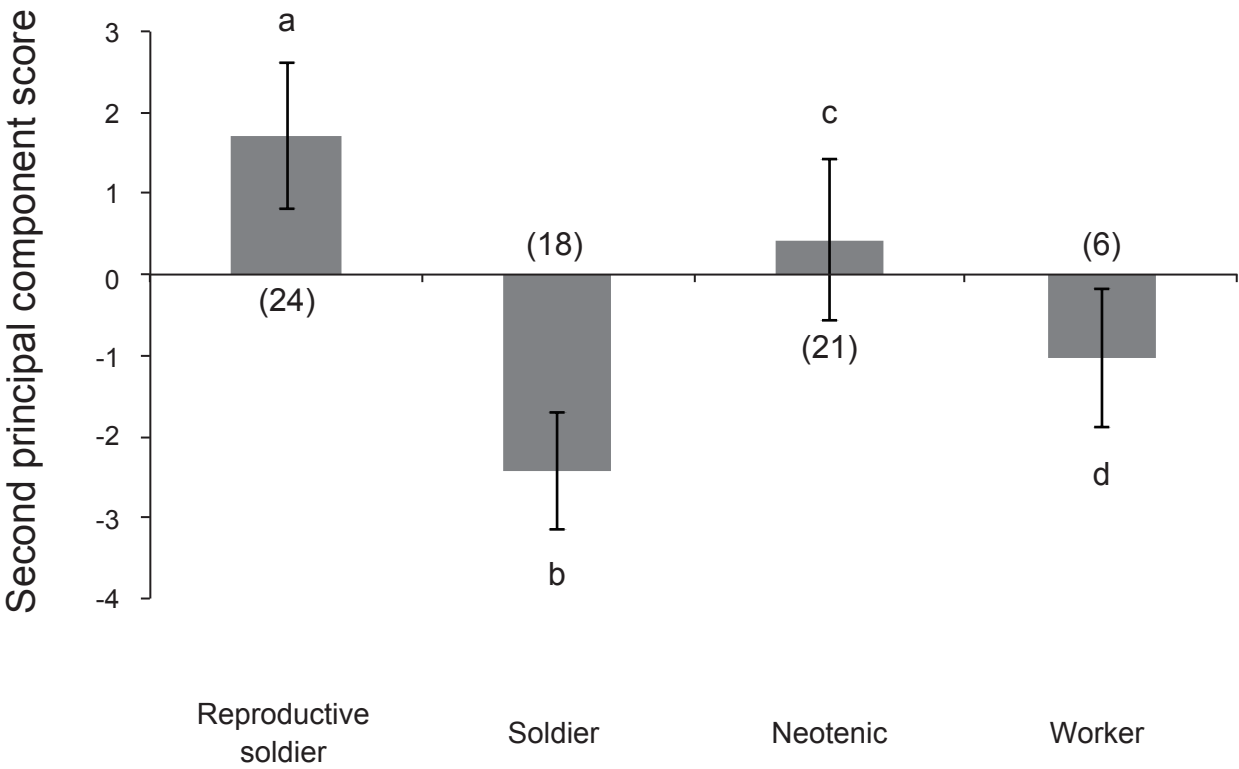

Fig. S2

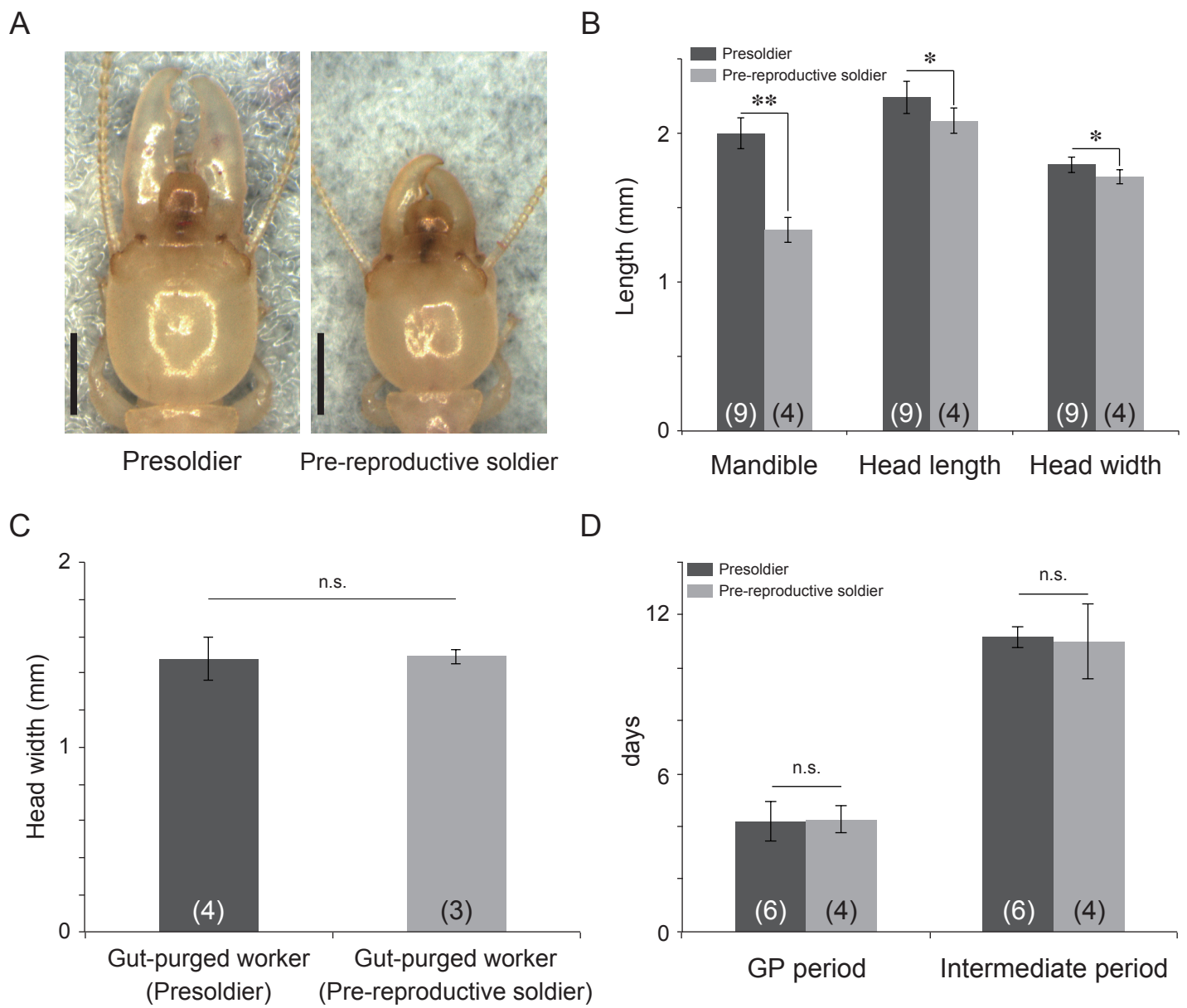

Fig. S3

Supplement: Supplementary file 1 [file insects-12-00076-s001.zip › supplementary_materials/SuppleFigs.pdf]
